# Seroprevalence and risk factors of epizootic hemorrhagic disease and bluetongue in Northwestern Tunisia: a comprehensive seroepidemiological study

**DOI:** 10.1186/s12917-025-05160-6

**Published:** 2026-01-24

**Authors:** Imed Ben Slimen, Sana Kalthoum, Aida Tlatli, Soufien Sghaier, Aida Megdich, Hanen Ncibi, Chafik Ben Salah, Ilyes Arfaoui, Mohamed yahya Dalhoumi, Marwa Sallami, Raja Gharbi, Kaoutker Guesmi, Sonia Ben Hsan, Salma Hadouchi, Mohamed Naceur Baccar

**Affiliations:** 1 Centre national de veille zoosanitaire, Tunis, Tunisia; 2 Food and Agriculture Organisation (FAO), Subregional Office for North Africa, les Berges du Lac 1, 1053 Tunis, Tunisia; 3 Institut de recherches vétérinaires, Tunis, Tunisia; 4 CRDA Beja, Beja, Tunisia; 5 CRDA Kef, Kef, Tunisia; 6 CRDA Jendouba, Jendouba, Tunisia; 7 Office des Terres Domaniales, Tunis, Tunisia

**Keywords:** Epizootic hemorrhagic disease, Bluetongue, Seroprevalence, Survey, Risk factors, Cattle, Tunisia

## Abstract

Epizootic hemorrhagic disease (EHD) and Bluetongue (BT) are vector-borne diseases that commonly circulate among wild and domestic ruminants. The epidemiological situation of EHD in Tunisia remains poorly documented, despite several studies having been conducted on BT. To assess the seroprevalence of the EHD and BT in northern Tunisia, 394 bovine serum samples were collected and tested for anti-VP7 antibodies using a competitive ELISA. The seroprevalence of EHD and BT at the individual level was estimated 51.2% and 81% respectively. Herd-level seroprevalence reached 93% for EHD and 100% for BT. No statistically significant differences in prevalence were observed between governorates for EHD (p-value = 0.169). However, the prevalence of BT across governorates was found to be statistically significant (p-value = 0.00000). A multivariable mixed-effects logistic regression was conducted to identify risk factors for EHD and BT. The final model revealed the only significant risk factor identified for both EHD and BT seroprevalence was age. Local animal husbandry practices and herd management were not found to be associated with the dynamics of the two diseases. The findings of this study highlight the geographical extent of the disease in the northwestern region and its associated risk factors. It is therefore imperative that further investigations be conducted on vectors and their abudance in order to gain a comprehensive understanding of the current situation.

**Supplementary Information:**

The online version contains supplementary material available at 10.1186/s12917-025-05160-6.

## Introduction

The genus *Orbivirus* comprises several viruses with significant impacts on animal health. Vector-borne diseases caused by viruses such as Epizootic hemorrhagic disease virus (EHDV) and Bluetongue virus (BTV) primarily affect livestock and wildlife, leading to severe outbreaks and significant economic losses for livestock. Both viruses belong to the family *Reoviridae* and share morphological and structural similarities [30]. The genome of both Orbiviruses consists of 10 double-stranded RNA segments encoding seven structural proteins (VP1 to VP7) and four non-structural (NS1–NS4) proteins. The VP7 protein, encoded by Segment 7, is highly conserved among serotypes of each virus and determines the BTV or EHDV serogroup. In contrast, the VP2 protein, encoded by Segment 2, is the principal neutralization antigen responsible for serotype specificity and is a major determinant of virus–host immune interactions ([2, 34, 61]. Classical serotypes of BTV and EHDV serotypes are capable of inducing clinical disease in domestic and wild ruminants and are listed by the World Organisation for Animal Health (WOAH) as notifiable diseases by the World Organization for Animal Health due to their significant animal health and trade implications [23]. Both viruses are responsible of a combination of similar clinical manifestations, including fever, serous or hemorrhagic nasal discharge, respiratory distress, cyanosis of the tongue, oral erosions and ulcers, lameness associated with coronitis, weakness, and mortality. In addition, affected animals may exhibit marked reductions in productivity [53].

Research has demonstrated how climate change exacerbates the distribution of such diseases, thereby enabling their emergence in regions where they were previously absent [14, 44, 54]. For instance, the spread of BT has been impacted by changing weather patterns affecting insect dynamics, thereby increasing the risk of outbreaks in susceptible animal populations [10]. BT and EHD are transmitted by *Culicoides* biting midges, and their co-circulation is considered a common phenomenon [54].

The EHD was detected for the first time in 1955 in North America [37]. According to literature, seven serotypes of EHD have been identified, numbered 1–2 and 4–8 [7]. BT was first recognized as a disease of cattle in 1933 [25]. As of now, 36 distinct serotypes of BTV have been identified, including 24 classical serotypes and 12 atypical serotypes [45, 46]. In comparison, EHDV comprises seven recognized serotypes [11]. Historically, BT has been recognized in the Mediterranean basin since 1924, with its African origin well-established [48]. EHD was first detected in Tunisia and other North African countries in 2006. Recent studies indicate persistent circulation of both BT and EHD in North African and Mediterranean countries. High seroprevalence of BT (36%−54%) was reported in 21 african countries [36]. In Mayotte, a cross-sectional study revealed high antibody prevalence in cattle for both BTV (99.5%) and EHDV (96.9%) between February and June 2016, suggesting widespread exposure [15]. In Kenya, seroprevalences for BTV and EHDV antibodies in calves aged 51 weeks were estimated at 94.2% and 63.7% respectively, demonstrating significant viral circulation [59].

The genetic diversity of BTV and EHDV in the Mediterranean region reflects their complex origins and spread. Phylogenetic analyses of BTV-2 from recent Mediterranean isolates show genetic similarity to strains from sub-Saharan Africa and North America, but distinctness from Asian strains (). Recent outbreaks caused by serotype 4 (BTV-4) detected in the Balearic Islands, Spain, in 2021, showing high genetic similarity to Tunisian strains, suggesting a possible introduction from North Africa [49].

Tunisia is endemic to both viruses. The first incursion of BT occurred in 1999, since then, multiple serotypes, including BTV-1, BTV-2, BTV-3, BTV-4, BTV-Y and BTV-26, have been identified [26]. The first outbreak of EHD was documented in 2006, resulting in significant economic losses for various cattle farms due to high mortality and morbidity. The Tunisian EHDV strains were classified as serotype 6 [6]. In 2021–2022, Tunisia experienced a large outbreak caused by EHDV-8 [57], presenting with symptoms similar to BT [7]. This serotype, originating from Northern Africa, has since spread to Italy (Sardinia and Sicily), Spain, and Portugal, highlighting rapid transboundary movement [43], Lorusso et al., 2023). Similarly, exceptional EHDV circulation was observed in France in 2023 [3], concurrently with BTV outbreaks [17].

While the epidemiology of BT has been relatively well studied in Tunisia over the past two decades with an overall seroprevalence of 40.1% reported and potential risk factors identified [26]. There is a lack of data on EHD, particularly in northwestern Tunisia, where no surveys have been conducted. This knowledge gap underscores the importance of assessing the seroprevalence of, and identifying risk factors for, both EHD and BT in this understudied region, especially given that a link between EHDV and BTV prevalence with vector distribution (*Culicoides imicola*) was highlighted in neighbouring countries [31].

The present study aims to assess the prevalence of EHDV and BTV antibodies in organized farms in northwest Tunisia. By identifying risk factors, it intends to provide valuable insights into EHD and BT transmission dynamics, contributing to effective disease management strategies.

## Materials and Methods

### Study area

**Fig. 1:**
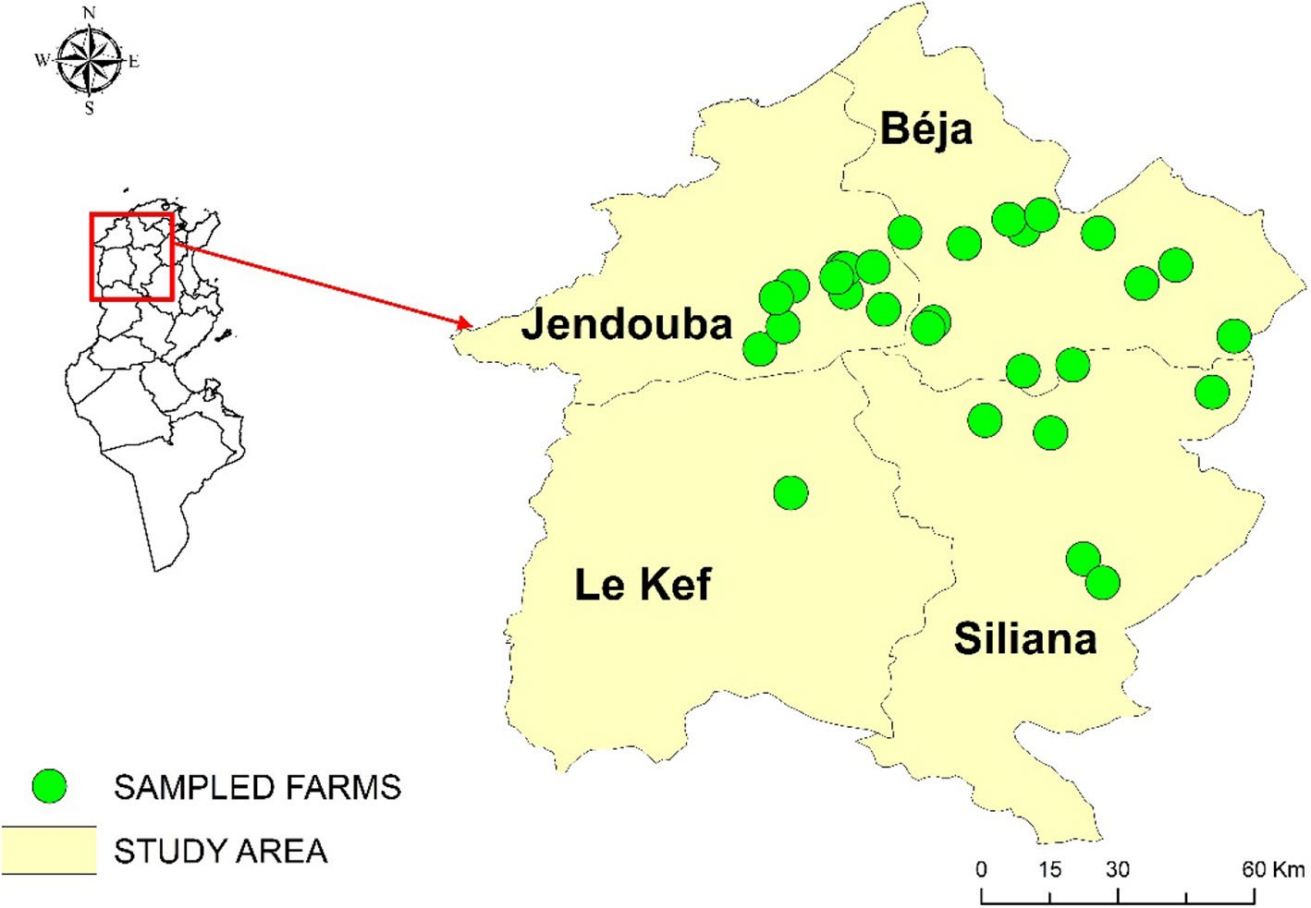
Study area and sampled farms

The study was conducted from 25 April to 19 May 2023, in four governorates in the north-western Tunisia: Jendouba, Beja, Siliana, and Kef (Fig. 1). These governorates were further subdivided into 76 districts and 744 sectors, covering an area of 16,267 km^2^, i.e. 10% of the total area of Tunisia with a total population of 1,210,000 inhabitants (Fig. 1). The northwestern region was selected for the study because it is the second most important agricultural area in Tunisia in terms of overall contribution to national activity. It is, however, the leading agricultural region in the country for red meat (40% with 38,200 tons), and milk (30% with 297,800 tons) (https://odno.nat.tn/). The cattle population in this region is estimated at about 152,023 head, mainly composed of local and crossbred dairy cattle (https://odno.nat.tn/). The study area is the rainiest region of the country, receiving more than 1,000 mm of rainfall per year. It is characterised by the presence of important watercourses and reservoirs.

### Farm selection and sampling design

The study population consisted of all organized farms in the north-western region of Tunisia, comprising 31 cattle farms from both the private and public sectors, with a total population of 8,634 cattle. As this is the first serological survey targeting EHD and BT in the region, all farms within the area were included in the study. The exhaustive list of animals across the 31 farms was provided by the regional veterinary services and the Office of Livestock and Pastures (OEP). No animals were euthanized or sacrificed during this study; only non-invasive blood sampling was conducted.

The following parameters were used to calculate of the required sample size:

- An expected prevalence of 25%, based on histrorical outbreaks data.
- A desired absolute precision of 15%
- A 5% risk of error.

The sample size (n = 484 animals) was determined using the Epi Info™ software (version 7.2, CDC, Atlanta, USA). A proportional sampling approach was used to determine the number of cattle to be sampled per farm, ensuring that the sample accurately reflected the distribution of animals across the farms. To guarantee representativeness of our sample, the cattle to be sampled were randomly selected using the sampling frame. Only animals over six months of age were included in the study to avoid potential interference with passive immunity. It is important to note that the study exclusively enrolled cattle, a species that has never been vaccinated against bluetongue (BT). In Tunisia, the BT vaccination program, previously limited to sheep, was discontinued in 2017 due to the circulation of multiple BTV serotypes, which complicated the selection of an effective vaccine strain. This ensured that all detected antibodies resulted solely from natural infection. For EHD, commercial vaccines have been employed in Europe since 2024. However, it should be noted that in 2023, in the region of the study, no vaccine was available. Therefore, this investigation provided an opportunity to assess the natural prevalence of EHD in cattle populations unexposed to vaccination. During the study, two farms initially scheduled to participate declined, reducing the final sample size to 396 cattle distributed across 29 farms.

### Cattle and farm characteristics

A total of 394 cattle, consisting of 386 females (97.9%) and 8 males (2.03%) belonging to 29 farms in four north-western governorates, were recruited for this study. As for the demographic characteristics of the sample, the age of the cattle ranged from 7 to 123 months with a mean of 40 months. The breed of the sample was predominantly Holstein constituting 85.02% (335/394) of the total. Other breeds including Montbeliarde, Swiss and Tarentaise accounted for 14.84% (59/394). For the gender, the sample is mainly of females accounting for 97.9% (386 out of 394). In term of age, the animals are categorised into three distinct age classes: 6–12 months, 13–24 months and over 25 months. The age group most represented is that of cattle over 25 months of age, representing 67.5% (266/394) of the sampled animals. Sample collection and laboratory testing.

Ten milliliters (10 mL) of blood was collected from each animal in strict adherence to animal welfare standards. Each sample was distinctly labeled using a predefined code accurate identification. All the samples were centrifuged to separate the serum and then, stored at −20 °C. The stored samples were subsequently transported under controlled conditions to the Institute of Veterinary Research of Tunisia (IRVT) for serological analysis. All sera were tested using the competitive enzyme-linked immunosorbent assay (ELISA) for EHDV (ID Screen® EHDV Competition, IDVet, France) and the BTV ELISA to detect antibodies directed against the VP7 protein (c-ELISA) kit (ID SCREEN® Bluetongue Competition ELISA, IDVet, France). Analyses were performed according to the manufacturer's instructions.

For each sample, we calculated the competition pourcentage (S/N% sample to negative ratio) using the Formula indicated in the manufacturer's instructions of ID Screen BT competition kit. Samples showing an SN% strictly lower than 40% are considered positive for antibodies against BT virus. Sampling showing an SN% equal or greater than 40% are considered negative.

Additionally, for each sample, we calculated the competition pourcentage (S/N% sample to negative ratio) using the Formula indicated in the manufacturer's instructions of ID Screen EHDV competition kit. Samples showing SN% greater than or equal to 40% are considered negative, greater than 30% and less than 40% considered doubtful and Less than or equal to 30% are considered positive for antibodies against EHDV.

### Data collection and statistical analysis

The study was carried out between April and May 2023. Farms and animals data were collected using a structured questionnaire specifically developed for this study consisting of three main parts. The first part focused on farm-related information, including the farm name, geographical location, type of building and housing conditions, total number of cattle, and the history of BT-like diseases on the farm. The second part is dedicated to individual animals and collected details such as age, sex, and breed. The final section covered potential risk factors associated with exposure to the BT and EHD, such as the introduction of animals, the presence of stagnant water, and proximity to wetlands. An English version of the questionnaire is provided as Supplementary File S1. Data was collected using an application of the Kobotoolbox platform(www.kobotoolbox.org).

For the environmental risk factors, data on temperature were extracted from https://www.ogimet.com for the period ranged between 30 march 2020 and 31 may 2023.

The extracted data was organized into an Excel spreadsheet. The age variable was transformed into a categorical variable with three defined age classes: Class 1(6–12 months), Class 2 (13–24 months) and Class 3 (more than 24 months). Herd size was categorized into three classes using the quartile classification method [50].

The seroprevalence of BTV and EHDV was estimated at both the herd and individual levels and the 95% confidence interval (CI) was calculated using the binomial exact method. The apparent herd prevalence was calculated as the proportion of positive herds among the total number of herds tested. The prevalence at the individual level was determined by dividing the number of positive animals by the total number of animals tested in that herd.

Raster for temperature was created using the interpolation method and values on farm location were extracted using Arcgis function “extract values to point”.

Raster for NDVI (Normalized Difference Vegetation Index) was extracted from https://www.copernicus.eu/en for the period between 30 april 2019 and 31 may 2023. Values on farm location were also extracted using Arcgis function “extract values to point”.

Univariate and multivariable mixed-effects logistic regression were conducted to identify risk factors for EHD and BTV at herd and animal level. The status of EHD and BT infection was designated as the outcome variable. The explanatory variables considered for univariate and multivariate analysis, are as follows: history of BT-like diseases on the farm, type of building, distance from wetlands, presence of Culicoides at the farm, presence of stagnant water on the farm, animal exchange with other farms, presence of wetlands close to the farm, age, sex, herd size, and breed. Associations between seroprevalence of EHD and BT and potential risk factors were initially assessed through univariable analysis and only variables with p < 0.2 were subsequently included in the logistic multiple-regression model. Odds ratios (ORs) and their 95% confidence intervals (95% CIs) were calculated. The presence of multicollinearity among the variables was evaluated using generalized variance inflation factors (GVIFs). The Hosmer–Lemeshow goodness-of-fit test was performed on the final model. Furthermore, receiver operating characteristic (ROC) curves and area under the curve (AUC) values were calculated in order to assess the model’s predictive performance.

All statistical analyses were performed using R software (version 4.3), and the maps were generated by ArcGIS version 10.4.

## Results

### Seroprevalence of the EHD and BT

**Fig. 2:**
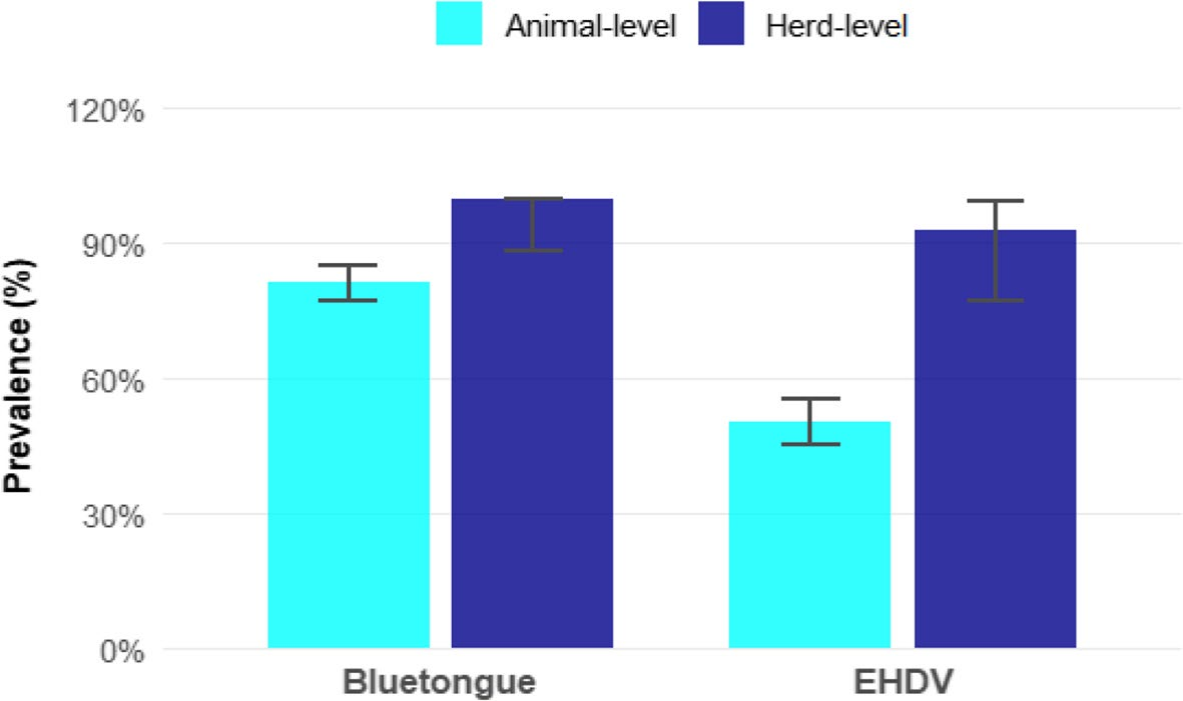
Prevalence of EHD and BT at the herd and animal level

At the herd level, all the farms were tested positive for the BT (29/29 (100%)) while 93.1% (95% CI: 77.23–99.15%) (27/29) were tested positive for anti-EHDV antibodies. At the individual animal level, 198 out of 394 samples tested positive for EHD giving an overall seroprevalence rate of 50.3% (95% CI: 45.2%−55.3%). For BT, 81,2% (320/394) (95% CI: 77%−85%) of the tested animals were positive (Fig. 2).

**Fig. 3:**
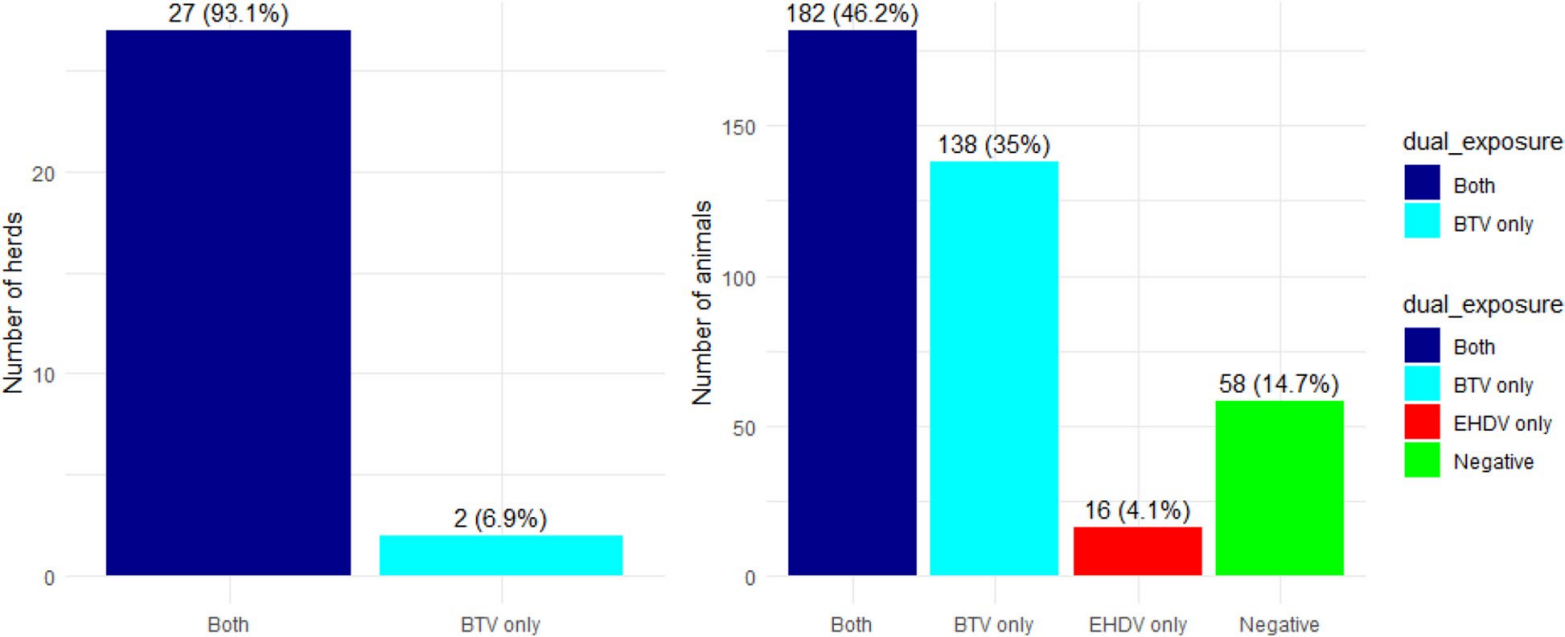
Dual exposure to EHD and BT at the herd and animal level

The presence of dual exposure to BT and EHD was detected in 27 (93.1%) of the tested herds. At the individual level, dual exposure to BT and EHD was detected in 182 (46.2%) of the total number of cattle (Fig. 3).

**Fig. 4:**
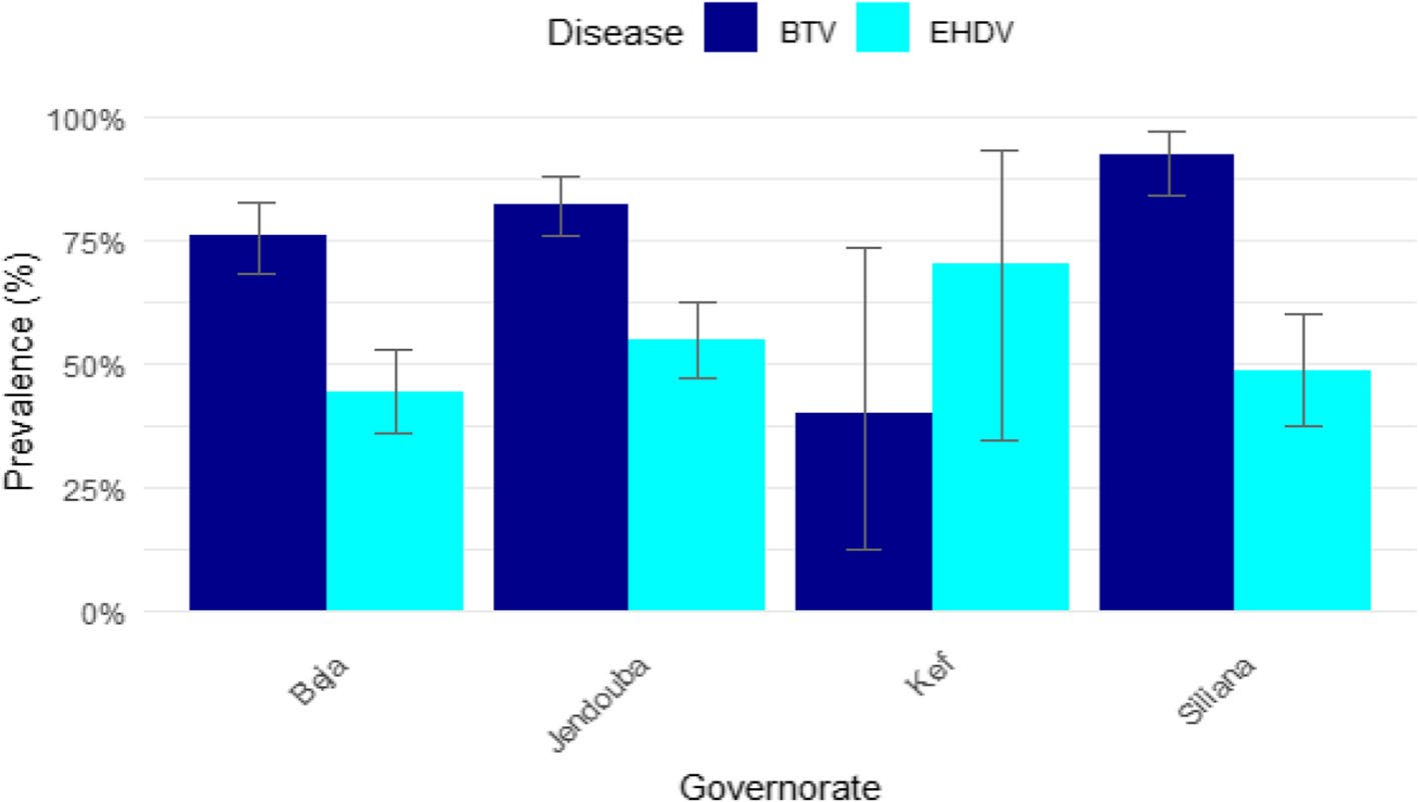
Seroprevalence of EHD et BT according to governorate

Seropositivity was assessed according to age group. Among young cattle (< 13 months), 16.1% [5.5–33.7%] tested positive for EHD and 71.0% [52.0–85.8%] for BTV. In the intermediate age group (13–24 months), 36.1% [26.7–46.4%] were seropositive for EHD and 67.0% [56.9–76.1%] for BT, while in older animals (> 24 months), seroprevalence reached 59.4% [53.3–65.3%] for EHD and 87.6% [83.0–91.3%] for BT. According to the governorate, 7 cattle out of 10 selected from the Kef governorate, were found to be positive to EHD representing the highest level of the seropositivity followed by Jendouba where 54.8% (91/166) of the cattle tested were positive. The lowest seroprevalence of EHD was recorded in Beja with 44.2% (61/138) of the cattle being seropositive. However, statistical analysis revealed no statistically significant differences between the governorates (p-value = 0.169). However, for BT, the results indicate that Siliana has the highest prevalence of BT with 92.5% (74/80), followed by Jendouba (82.5% (137/166), Beja (76.1% (105/138) and Kef (40% (4/10)) (Fig. 4). The observed variation in the prevalence of BT across governorates was found to be statistically significant (p-value = 0.00000)..

### Risk factors of EHD and BT

**Fig. 5:**
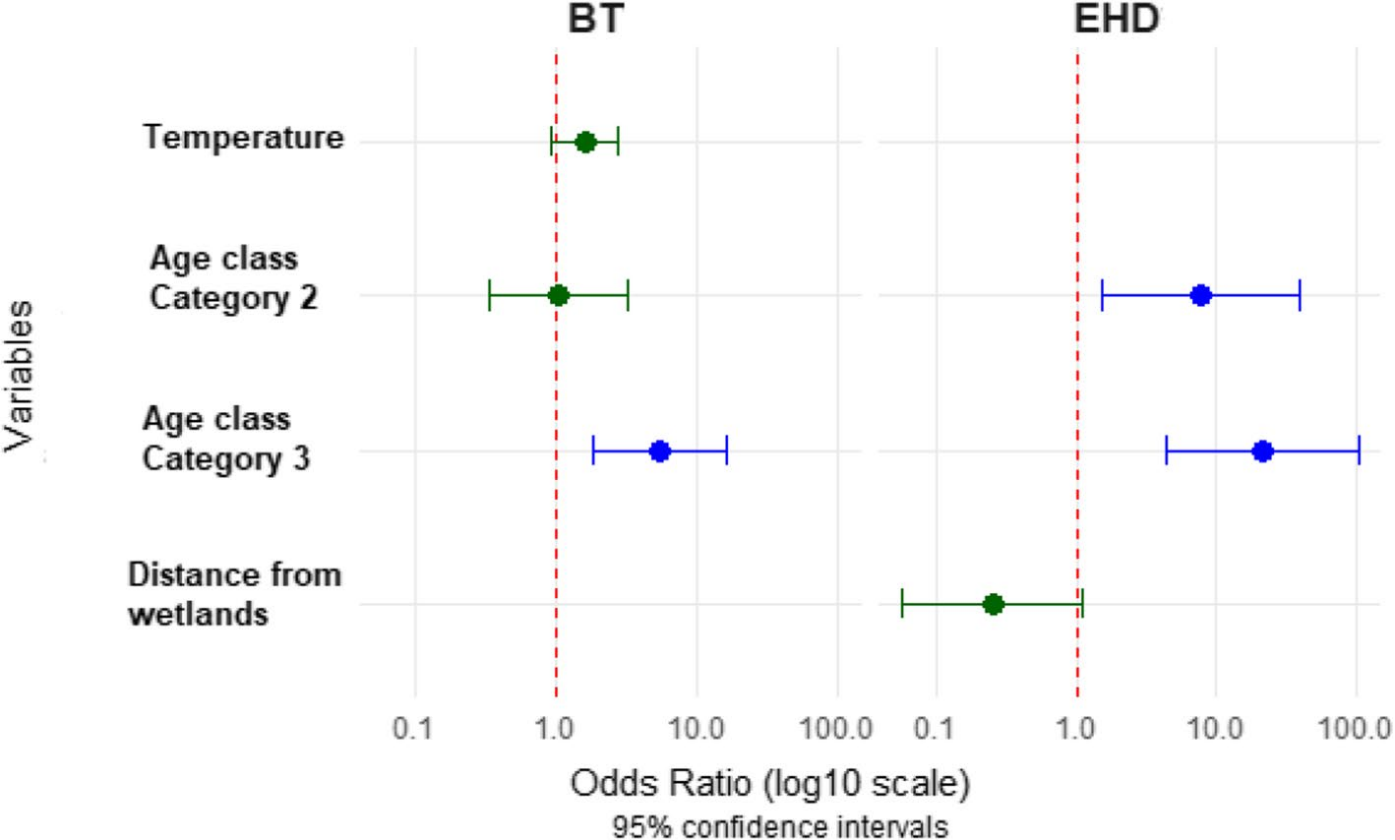
Potential risk factors associated with BT and EHD seropositivity among cattle in Northwestern Tunisia

Univariate analysis revealed that the age and temperature were identified as significant predictors of BT. As demonstrated in Table 1, the risk factors associated with a positive test result for EHD were distance from wetlands, age and the presence of wetlands (Table 1). The final model demonstrated that the age was the only significant predictor for both EHD and BT. Animals aged 25–123 months (Category 3) have significantly higher odds of seropositivity of BT compared to younger (OR = 5.30, 95% CI: 1.77–15.87, *p* = 0.002). While temperature showed a non-significant association with BT seropositivity (OR = 1.57, 95% CI: 0.91–2.73, *p* = 0.10). Regarding EHD, both category of age (Category 2 and 3) demonstrated significantly increased odds compared to younger animals (Category 2:12–24 months; OR = 7.81, 95% CI: 1.54–39.52, p = 0.01, Category 3:25–123 months; OR = 21.1, 95% CI: 4.38–102.11, *p* = 0.0000). Farms located less than 1 km from wetlands tended to have lower odds of EHD seropositivity, although, this association did not reach statistical significance (OR = 0.25, 95% CI: 0.059–1.11, *p* = 0.07) (Table 2, Fig. 5). All other variables, including housing type, breed, Culicoides presence in the farm, NDVI, stagnant water, and flock size, showed no significant associations with either disease in the univariate analysis.

**Table 1:** Univariate analysis for the association between potential risk factors and BT and EHD seropositivity among cattle in Northwestern Tunisia

Variables	Category	BT		EHD	
		OR [IC95%]	***p***-value	OR [IC95%]	***p***-value
Housing type	Semi-enclosed	1.44 [0.06–34.42]	0.82	1.62 [0.09–28.14]	0.74
Breed	Holstein	0.63 [0.15–2.70]	0.54	0.95 [0.29–3.09]	0.93
Distacne from wetlands	Distance < 1 km	0.59 [0.17–2.05]	0.41	0.26 [0.06–1.02]	0.05
Presence of Culicoides in the farm	Yes	0.51 [0.07–3.74]	0.51	0.81 [0.15–4.36]	0.81
NDVI	NDVI (per unit)	1.07 [0.05–22.49]	0.97	0.85 [0.06–11.08]	0.9
Presence of stagnant water	Yes	1.46 [0.15–14.53]	0.75	1.03 [0.13–8.20]	0.98
Age class	Category 2: [12–24 moths]	1.02 [0.33–3.14]	0.98	7.02 [1.98–24.87]	0.002
	Category 3: [25–123 months]	5.31 [1.77–15.87]	0.003	20.20 [5.91–69.03]	0.000
Flock size	[> 300]	0.72 [0.20–2.54]	0.61	0.92 [0.30–2.78]	0.88
Temperature	Temperature (per unit)	1.52 [0.94–2.47]	0.09	1.25 [0.79–1.97]	0.35

**Table 2:** Multivariate analysis for the association between potential risk factors and BT and EHD seropositivity among cattle in Northwestern Tunisia

Variables	Category	BT		EHD	
		**OR [IC95%]**	***p*-value**	**OR [IC95%]**	***p*-value**
Distacne from wetlands	Distance < 1 km	-	-	0.25 [0.059–1.11]	0.07
Age class	Category 2: [12–24 moths]	-	-	7.81 [1.54–39.52]	0.01
	Category 3: [25–123 months]	5.30 [1.77–15.87]	0.002	21.16 [4.38–102.11]	0.0001
Temperature	Temperature (per unit)	1.57 [0.91–2.73]	0.10	-	-

## Discussion

In Tunisia, the surveillance system of vector-borne diseases such as BT and EHD relies only on the passive surveillance. Since the first report of EHD in Tunisia and according to the previous study, the northwestern region has been identified as a highly infected area [56]. Both diseases (BT and EHD) can be transmitted by the same vector and cases of co-infection have been previously documented [7]. To our knowledge, few studies have been considered the seroprevalence of the vector-borne diseases specially in the northwestern Tunisia. Therefore, the present study aims to assess the serporevalence of BT and EHD in organized farms in the northwestern Tunisia and to identify potential risk factors.

The results of this study indicate the widespread of the occurrence of BT and EHD in the northern Tunisia. The overall seroprevalence of EHD estimated animal-level in this study (50.3%) is considerably higher than that reported in previous studies. Mejri et al. 37, reported a seroprevalence of 8.6% during a sentinel study conducted in cattle between 2013 and 2014, Ben Dhaou 5 detected a seroprevalence of 5.3% in the governorate of Bizerte in 2012. This notable increase in seroprevalence may reflect a recent intensification in virus circulation and can be attributed to several factors such as favorable climatic conditions for vector activity and changes in vector competences, abundance and distribution [18]. It was demonstrated that significant changes have been observed in the pattern of disease and distribution of EHDV and an increase in the severity of the disease among bovine populations [23, 41]. At the regional level, our findings contrast with those reported in neighbouring North African countries, where EHDV seroprevalence remains relatively low. Studies in Libya (2021) and Algeria (2011) reported seroprevalences of 1% and 9% in cattle, respectively while a prevalence of 10% was recorded in India in 2010 [9, 31, 33]. Conversely, our results align with the findings of various authors across different countries: 57.87% in China, 57.1% USA (Northern Florida), 66.9% in Pakistan and 64% in Kenya [19, 22, 29, 42].

For BT, the overall seroprevalence at the individual level was very high (81.2%) in our study indicating a high degree of exposure of cattle in the northern Tunisia to the virus. This result suggests recent circulation of the virus of BT in the study area. The observed high seroprevalence in these farms is likely indicative of a substantial population of Culicoides vectors that is facilitated by favorable climatic conditions. As demonstrated in the relevant literature, comparable high seroprevalences of BT have been reported in Kenya (91.5%), Estern Sudan (92.9%) and Brazil (83.3%) [1, 13, 39]. However, the seroprevalences of BT reported in southern Italy (43.6%), Iran (27.63%), Algeria (13.7%) and Peru (19.3%) were lower than our finding [4, 16, 27, 38]. When compared to these regional and international findings, the high BT seroprevalence observed in northern Tunisia may indicate both historical virus persistence and ecological suitability for sustained vector transmission, underscoring the need for continued entomological and serological surveillance in this region.

The present study indicated that 46.2% of the tested cattle were co-infected with both BT and EHD. This is considered a high prevalence in comparison to the studies conducted in Mayotte and USA, where 9.4% and 1.49% of the samples were positive for both diseases, respectively [52, 55]. However, it aligns with the findings of Min-Na Lv et al. in China in 2012–2013 (40.3%) [29]. The presence of BT and EHD coinfections observed in our study can be attributed to several factors. The viruses that cause theses diseases are both transmitted by the same genus of hematophagous biting midges (Culicoides spp.) and when the ecological niches of the two diseases overlap [29]. The climatic conditions the study area that are characterized by cold, rainy winters and hot, dry summers, create an ideal environment for vector survival and reproduction, potentially extending the transmission season and increasing the risk of infection. Warm temperatures promote breeding, feeding, and virus replication, while, rainfall increases breeding sites by maintaining moist soil, preventing desiccation, and thus increasing vector population size [24]. Consistent with other research [9, 47], our results confirm that significant exposure to EHDV and BTV is associated with the abundance of Culicoides spp. in the region.

The high seroprevalence of both disease observed in our study reflects intense and sustained virus circulation, consistent with the recent emergence of these viruses across the Mediterranean region. Molecular evidence confirms a strong epidemiological linkage between North Africa and Southern Europe: EHDV-8 strains detected in Tunisia in 2021–2022 share high nucleotide identity with those subsequently identified in Italy [28] and red deer in Spain [51]. Similarly, BTV-4 strains circulating in the Balearic Islands in 2021 showed close genetic relatedness to Tunisian isolates [49], supporting the hypothesis of a northward spread from North Africa and the epidemiological connectivity driven by geographical proximity.

The study revealed no significant differences in the EHD seroprevalence across governorates suggesting uniform exposure of cattle to this disease in the study area, wich may be due to similar environmental conditions and vector abundance. However, a significant variation in the seroprevalence of BT was highlighted, wich can be attributed to the differences in livestock species composition, breed susceptibility, and host and vector density [12, 53]. Additionally, the seroprevalence of BT may be influenced by herd management practices, including vector control measures and grazing patterns [8].

Based on the univariate and multivariate analysis, we studied the association between seroprevalence of BT and EHD and the following risks factors; Housing type, breed, distance from wetlands, presence of mosquitos in the farm, NDVI, presence of stagnant water, age class, flock size and temperature. The results revealed that only the age is a risk factor for both diseases in the final model, but with stronger effects observed for EHD, reflecting distinct epidemiological dynamics between the two infections. The age-related increase in seroprevalence observed for both viruses aligns with findings from previous studies, which attribute higher exposure over time to the cumulative effect of vector contact as cattle age [13, 21, 36, 57]. This highlights the endemic nature of these pathogens in environments with a high prevalence of vectors.

The absence of significant breed-specific differences in seroprevalence of the two vector-borne diseases, previous studies conducted overwide suggest that susceptibility to EHD and BT in endemic areas is primarily determined by vector exposure rather than genetic factors [35, 58]. Unexpectedly, housing type, presence of stagnant water, and NDVI were not significantly associated with seroprevalence of either disease. First, Culicoides are indoor and outdoor vectors and seasonal variation in climatic conditions influences their indoor and outdoor activity patterns [20, 32]. Second, Culicoides midges may breed in moist soils or microhabitats not captured directly by broad environmental indicators such as NDVI or visible stagnant water bodies [40, 60]. The other investigated risk factors were found to be significantly associated with either disease.

Despite the valuable data provided by this study, several limitations should be acknowledged. First, the study relied on cross-sectional serological data, which limits the ability to ascertain whether the viruses under investigation are recent or old circulation. Furthermore, environmental data used in this study such as temperature and NDVI were extracted from external sources and did not cover all the time periods of exposure, which may influence the association of these variables with BT and EHD seroprevalecne. Finally, the study did not include molecular detection of active infections or virus typing, which could provide deeper insights into circulating strains and the dynamics of co-infections.

The findings of the present study enhance our comprehension of the epidemiological profiles of two vector-borne diseases (BT and EHD) in northern Tunisia, as well as the risk factors associated with their seroprevalences. The study provides valuable information for the control programmes of these diseases and for the adaptation of strategies in order to reduce the risk of their occurrence.

## Conclusion

The present study highlights the widespread of occurrence of BT and EHD in organised cattle farms across the northwestern Tunisia. The high seroprevalence of both diseases indicates favourable environment for the vectors implicated in their transmission and spread. Although the study provided valuable data on the seroprevalence of the two diseases, it remains unclear whether these infections were occurred. Future longitudinal studies incorporating molecular diagnostics and entomological surveillance are needed to better understand the transmission dynamics and temporal trends of BT and EHD in Tunisia.

## Supplementary Information

Supplementary Material 1.

## Data Availability

The datasets generated and analyzed during the current study are available from the corresponding author on reasonable request.
