# Supplementary material for: Seroprevalence and risk factors of epizootic hemorrhagic disease and bluetongue in Northwestern Tunisia: a comprehensive seroepidemiological study
Source: BMC Vet Res. 2026 Jan 24;22:151. doi: 10.1186/s12917-025-05160-6 (PMC12955279; doi:10.1186/s12917-025-05160-6)
Supplement: Supplementary file 1 — Supplementary Material 1. [file 12917_2025_5160_MOESM1_ESM.pdf]

Centre National de Veille Zoosanitaire<br>المركز الوطني لليقظة الصحية الحيوانية	**QUESTIONNAIRE FORM**<br>***EHDV/BT***	 **Version : April 2023**

I- GENERAL INFORMATION :

Gouvernorate : ………………Delegation :………………………Imada : ……………….
Date: ……./………/…………
Conducted by: …………………………..Phone : ……………. …..Fax :……………

II- SPECIFIC INFORMATION :

1- Farm identification :

Farmer's Name : ……………………………….. Phone: …………………………………
GPS Coordinates: X: …………………………. Y : ……………………………..

Housing Type : ☐ Enclosed Housing ☐ semi-open Housing ❑ Others
If other, please specify : ………………………………………………………………

2- Renseignements sur les espèces présentes dans l'élevage :

Species	Number of Animals	
	>6 Months	< 6 Months
cattle		
Sample		

Disease :

Presence of symptoms suggestive of EHDV/BT: ❑ Yes ❑ No
If Yes, year of onset: ……………………………………..
Number of sick animals : ……………………Number of deaths : ………………………..
Number of abortions : ……………………………………………………………………….

Observed clinical signs :

- ❑ Febrile syndrom
- ❑ Oral congestion with hypersalivation (petechiae, ulcers)
- ❑ Facial edema
- ❑ Discharge (nasal, ocular)
- ❑ Eye lesions (Conjunctivitis)
- ❑ Boiteries ou gonflement des membres
- ❑ Respiratory disorders
- ❑ Hemorrhagic diarrhea
- ❑ Drop in milk yield

Progression of clinical signs: ❑ Recovery ❑ Complication ❑ Death
Use of treatment: ❑ Yes ❑ No

3- Risk Factors :

a- Introduction :

New Introduction: ❑ Oui ❑Non
If yes, specify since when :………………..

b- Surroundings :

Presence of wetlands near the farm : ❑ Yes ❑ No Distance (km) : …………
Wetland type: ……………………………………………………………
Presence of similar clinical symptoms in neighboring farms: ❑ Yes ❑ No
Grazing near wetlands: ❑ Yes ❑ No

Other Risk Factors :

Presence of mosquitoes in the farm: ❑ Yes ❑ No
Presence of stagnant water in the farm: ❑ Yes ❑ No
Animal exchanges with other farms: ❑ Yes ❑ No

Centre National de Veille Zoosanitaire<br>المركز الوطني لليقظة الصحية الحيوانية	***SAMPLE COLLECTION FORM***	**Version : April 2023**

Samples collected by: …………………………Phone : ……………..Fax :……………

Adress : ………………………………… Date of sampling: ……../……../………..

Farmer's Name : ………………………………….Phone : ……………………

Gouvernorate : ………………Delegation :………………………Imada : ………………

	**Identification Number**	**Breed**	**Sex**	**Age (Months)**
1				
2				
3				
4				
5				
6				
7				
8				
9				
10				
11				
12				
13				
14				
15				
16				
17				
18				
19				
20				
